# Multiple paragangliomas diagnosed in head, neck, and mediastinum: a case report

**DOI:** 10.1186/s13000-025-01710-6

**Published:** 2025-09-30

**Authors:** Shahab Rafieian, Hesam Amini, Omid Rezaei, Aysan Nozheh, Niloofar Ayoobi Yazdi

**Affiliations:** 1 Department of Thoracic Surgery, Imam Khomeini Hospital Complex, Tehran University of Medical Sciences, Tehran, Iran; 2 Department of General Surgery, Imam Khomeini Hospital Complex, Tehran University of Medical Sciences, Tehran, Iran; 3 Department of Pathology, Cancer Institute, Imam Khomeini Hospital Complex, Tehran University of Medical Sciences, Tehran, Iran; 4 Department of Radiology, Imam Khomeini Hospital Complex, Tehran University of Medical Sciences, Tehran, Iran

**Keywords:** Case report, Neuroendocrine tumors, Paraganglioma, Surgical management

## Abstract

**Background:**

Paragangliomas are neuroendocrine tumors that often present as solitary tumors. In this case report, we describe a patient with multiple head and neck paraganglioma associated with a mediastinal paraganglioma.

**Case presentation:**

The patient was a 46-year-old male with a history of surgical removal of a mass from the right side of the neck, who presented with dysphonia lasting two months, hoarseness, vague chest pain, and unilateral ptosis. CT angiography of the carotid arteries and thoracic aorta revealed multiple findings, including a well-defined enhancing mass measuring 33 × 39 mm in the aorto-pulmonary prevascular space, a grade I carotid body tumor on the left side of the neck, vagal paragangliomas on the right side of the neck, and a glomus jugulare tumor on the right side. These findings were collectively suggestive of multiple paragangliomas. The patient subsequently underwent surgical resection of the mediastinal tumor, and pathological examination confirmed the diagnosis of paraganglioma.

**Conclusion:**

This report details a rare case of paraganglioma with multiple head, neck, and mediastinal involvement, emphasizing the need for thorough evaluation and genetic assessment in atypical presentations.

## Introduction

Paragangliomas are neuroendocrine tumors that arise from extra-adrenal sympathetic or parasympathetic ganglia and contain the cellular structures usually seen in paraganglions, including granular, and sustentacular cells [1–3]. With an incidence of 2 to 8 per one million people, paragangliomas are relatively rare [3]. Clinical manifestations of paragangliomas depend on their location and disease stage. They are typically nonfunctional and asymptomatic tumors that, due to their slow growth, often remain unnoticed until the later stages of the disease, when they present as a palpable mass. However, they can also present with symptoms related to catecholamine release, such as sweating, headaches, and tachycardia [4, 5]. Although these tumors rarely become malignant, their tendency for local invasion results in a high recurrence rate [5].

Accurate localization and identification of paragangliomas are crucial, as the tumor’s location significantly impacts the patient’s survival. Patients with abdominal, pelvic, or bladder paragangliomas tend to have worse outcomes and lower survival rates [6]. Additionally, the recurrence of paragangliomas depends on their anatomical location, with glomus jugulare tumors showing a higher likelihood of recurrence [7]. Paragangliomas can develop anywhere paraganglia are located, whether during embryonic development or in adulthood [8]. Despite this, the head and neck region is the most common site for paraganglioma diagnosis, accounting for up to 70% of all cases [5]. However, there have been reports of rare cases of paragangliomas occurring in unusual locations throughout the body [8].

The majority of paragangliomas are solitary tumors. However, a thorough evaluation of patients is crucial due to the increased risk of familial genetic disorders in the 10% of cases with multifocal disease. Additionally, these tumors may occur as part of hereditary syndromes such as multiple endocrine neoplasia, neurofibromatosis, and von Hippel-Lindau disease [9, 10]. Mutations in succinate dehydrogenase complex subunit D, hypoxia-inducible factor 2 A, endothelial pas domain protein 1, and malate dehydrogenase2 are suggested to increase the risk of multifocal paraganglioma [11]. However, there are only a few case reports describing the cases with multiple paragangliomas [12–16]. In this case report, we describe a patient with multiple head and neck paraganglioma associated with a mediastinal paraganglioma.

## Case presentation

The patient was a 46-year-old male who presented to our outpatient clinic at Imam Khomeini Hospital Complex, a tertiary hospital in Iran, with dysphonia, hoarseness, vague chest pain, and unilateral ptosis that had started two months prior to his referral. The patient had no other signs or symptoms, and apart from the dysphonia, the physical examination was unremarkable. The patient had no history of medical conditions, did not take any medications, and had no history of smoking or tobacco use. He reported a mass on the right side of his neck that was surgically removed four years ago but did not have access to the medical records from that procedure. Additionally, the patient mentioned that his cousin also had a history of a neck mass that was surgically removed. However, we were unable to access medical documents for either the patient or his cousin to determine the type of the masses. The patient had previously undergone both upper gastrointestinal endoscopy and colonoscopy before being referred to our center, with no significant findings reported in either procedure.

**Fig. 1:**
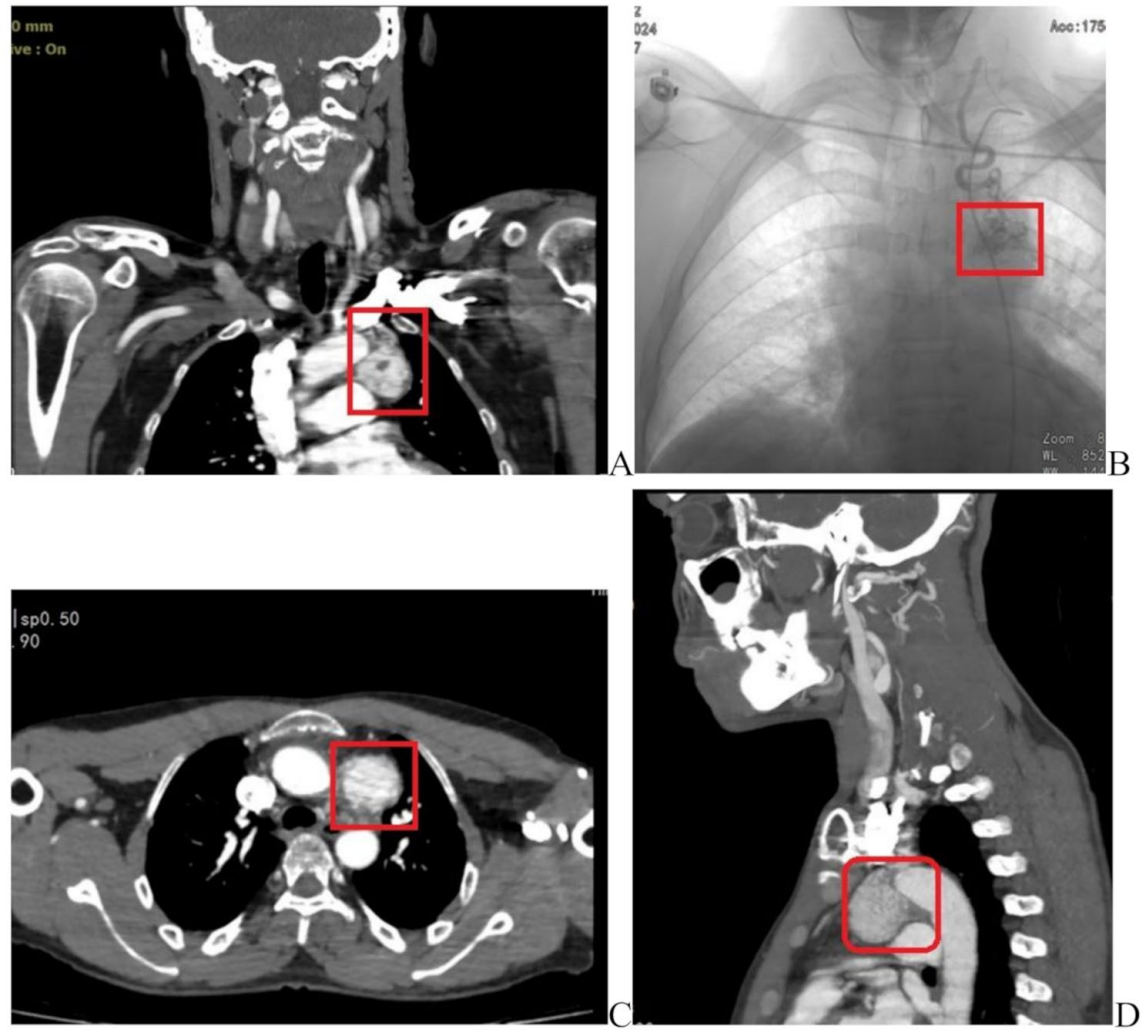
CT angiography of the aorta showing a mass in the aortopulmonary prevascular space

We performed computed tomography (CT) angiography of the carotid and thoracic aorta to further evaluate the patient due to suspicion of mediastinal and neck masses. In the CT angiography of the aorta, a well-defined enhancing mass measuring 33 × 39 mm with a craniocaudal length of 42 mm was identified in the aortopulmonary prevascular space. The fat plane between the mass and the aortic arch remains intact, with no evidence of invasion into the surrounding tissues (Fig. 1). In the carotid arteries CT angiography, a heterogeneous enhancing mass measuring 15 × 12 mm was observed at the left common carotid artery bifurcation, suggestive of a grade I carotid body tumor. Additionally, another heterogeneous enhancing mass measuring 12 × 8 mm was detected in the posterior carotid space on the right side, suggestive of vagal paragangliomas. This mass had displaced the internal carotid artery anteromedially and the jugular vein posterolaterally. Furthermore, there was a widening of the jugular foramen on the right side, accompanied by bone destruction, which was suggestive of glomus jugulare. There were no other pathological findings in the CT angiographies.

The CT angiographies primarily suggested the presence of multiple head and neck paragangliomas, associated with a mediastinal mass. To assess the patient for potential brain metastasis, a brain magnetic resonance imaging (MRI) with and without contrast was performed, which revealed no abnormalities. The patient then underwent surgical resection of the mediastinal tumor. The surgical procedure included median sternotomy due to the presence of a mass in the mediastinum. Intraoperatively, the mass was found to be adherent to both the thymus and the pericardium. The portion of the thymus overlying the mass was excised along with the tumor and sent for pathological evaluation. Additionally, a segment of the pericardium that was attached to the mass was resected. The resulting pericardial defect was repaired. The mass had several vascular connections to the aortic arch, which were carefully ligated using surgical clips. Two chest tubes were inserted: one in the mediastinum and another in the left hemithorax to ensure adequate drainage.

**Fig. 2:**
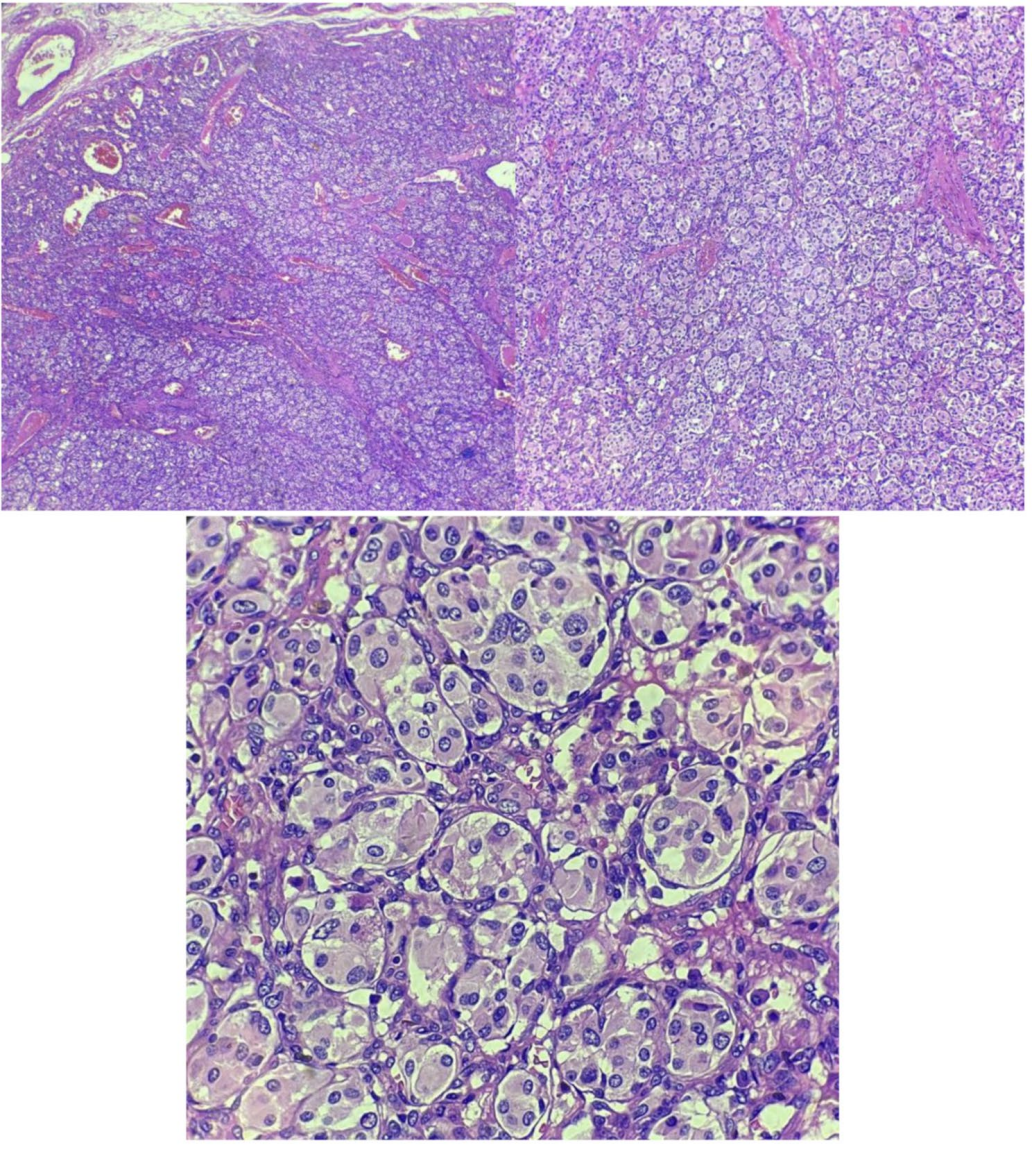
Histopathological examination of the mass (x40, x100, x400)

**Fig. 3:**
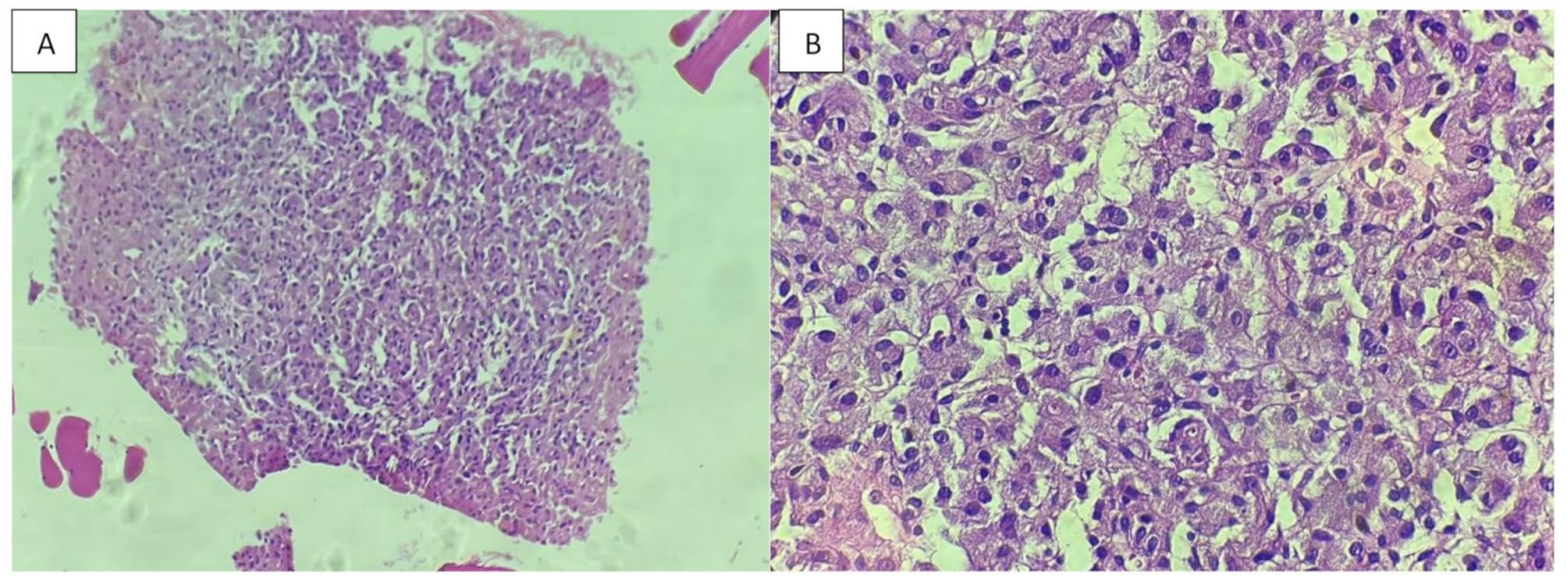
Biopsy specimen: Histological sections stained with hematoxylin and eosin (H&E); **A-B** (At magnification x40 and x400 respectively); The biopsy sections consist of fragments of tumor tissue composed predominantly of semi-nested and loosely cohesive clusters of polygonal epithelioid chief cells. These cells exhibit moderate amounts of granular eosinophilic to amphophilic cytoplasm and round to oval nuclei with finely granular chromatin and some inconspicuous nucleoli and a delicate fibrovascular stroma. No significant mitotic activity, necrosis, or hemorrhage is observed

**Fig. 4:**
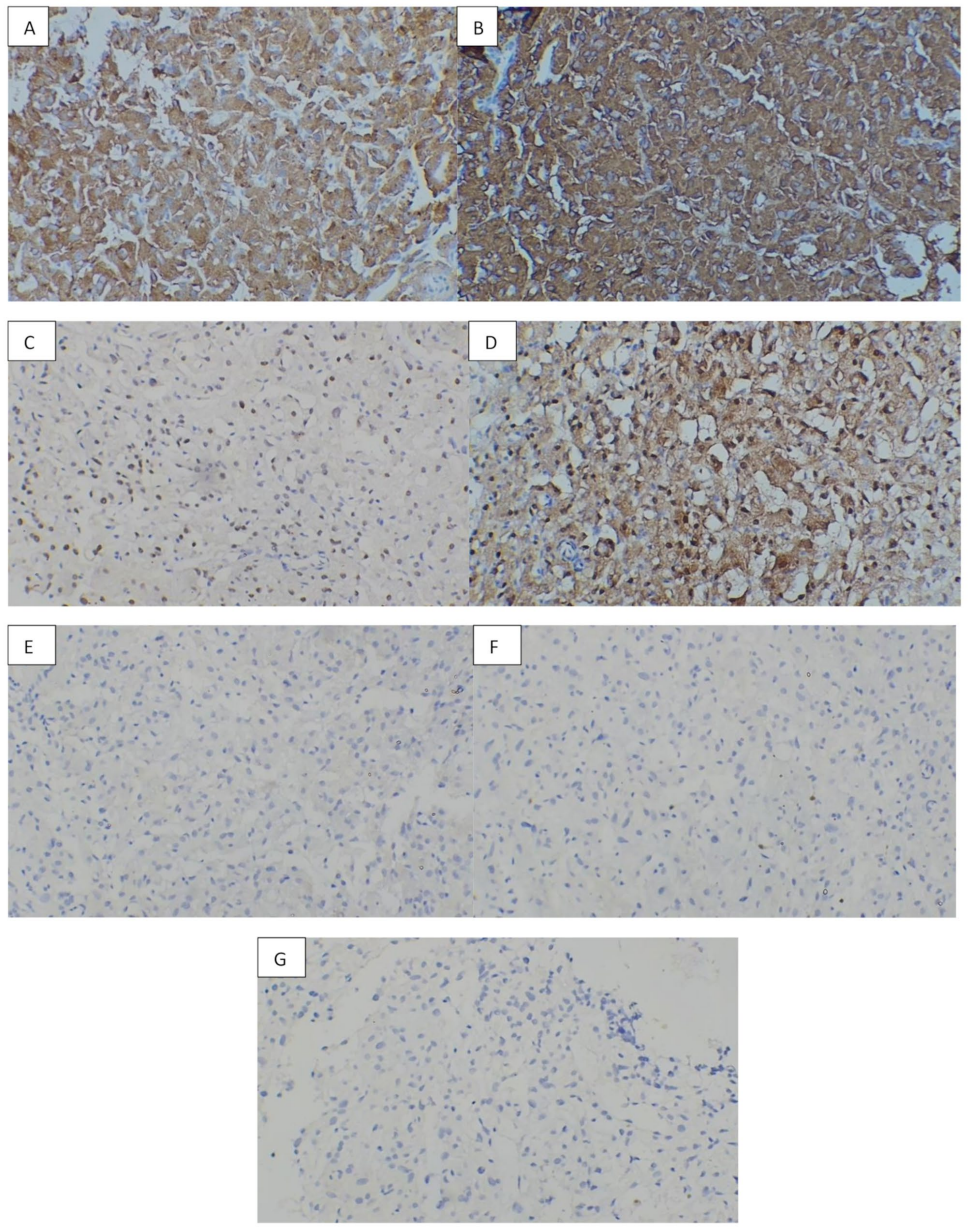
Immunohistochemistry (IHC) staining (At magnification x400); **A**: Synaptophysin, Positive, **B**: Chromogranin, Positive, **C**: GATA3, Positive, **D**: S100, Positive, **E**: Cytokeratin, Negative, **F**: Inhibin, Negative, **G**: Calretinin, Negative. IHC results are consistent with paraganglioma

**Fig. 5:**
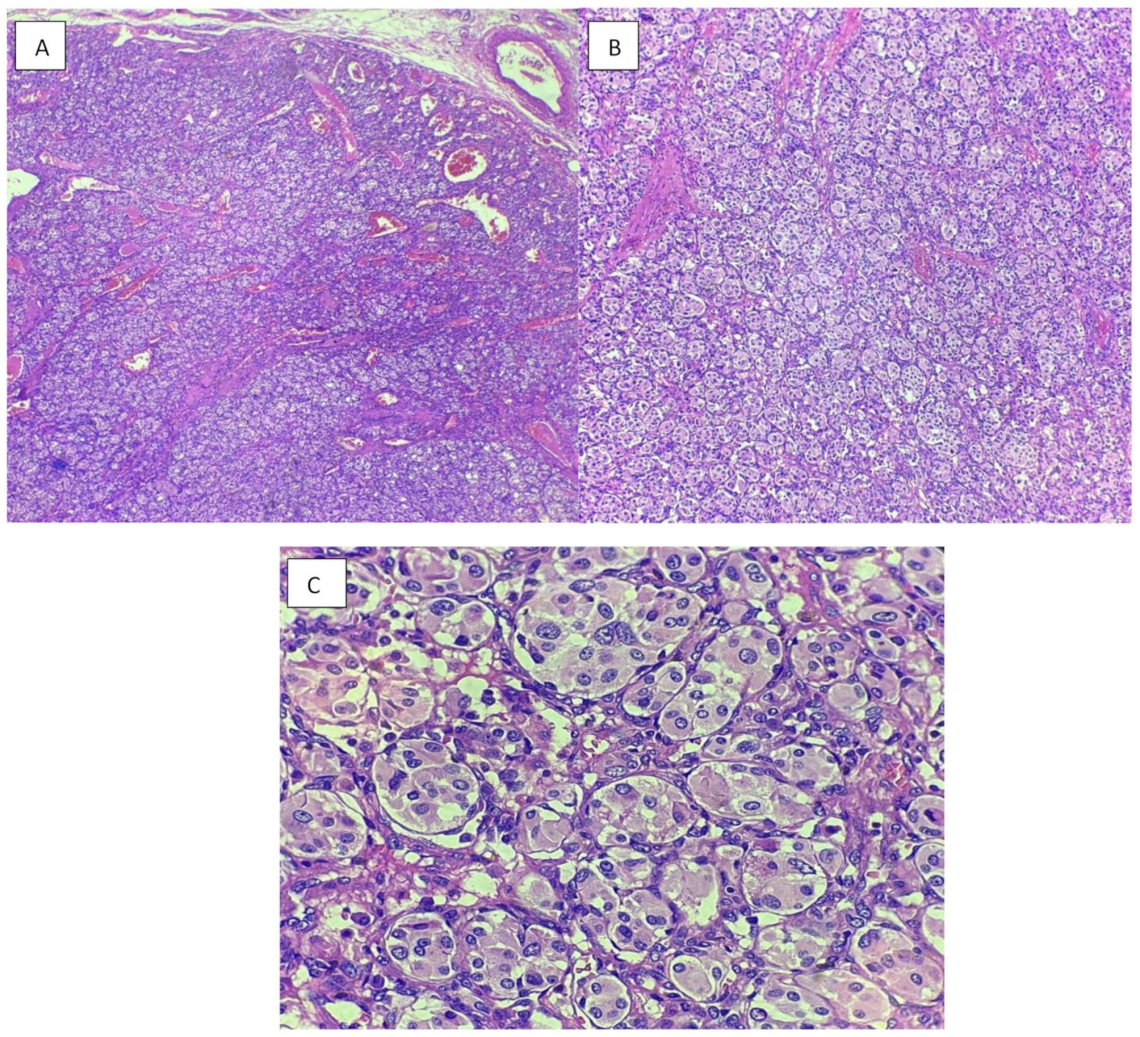
Resection specimen: Histological sections stained with hematoxylin and eosin (H&E); **A-C** (At magnification x40, x100 and x400 respectively); sections show the tumor is composed of well-defined nests (Zellballen pattern) of polygonal to round epithelioid chief cells with abundant granular eosinophilic cytoplasm. These nests are separated by a prominent, delicate fibrovascular stroma rich in thin-walled capillaries. The chief cells typically exhibit round to oval nuclei with finely granular (“salt and pepper”) chromatin and inconspicuous nucleoli. Occasional nuclear pleomorphism and rare mitotic figures is also seen

The resected mediastinal mass was sent for pathological examination (Fig. 2). The histopathological analysis confirmed the diagnosis of paraganglioma, with the greatest dimension measuring 3 cm. Additionally, the specimen showed the presence of intravascular foreign bodies related to angioembolization material, introduced during preoperative embolization (Fig. 3, 4, 5). Examination of the surrounding tissue revealed unremarkable thymic tissue, with no evidence of malignancy or other pathological findings. We were unable to perform genetic testing due to limited resources.

The patient was admitted to the intensive care unit (ICU) for one week and subsequently discharged from the hospital. No postoperative complications, especially catecholamine-related ones, were observed. During the follow-up three months after surgery, the dysphonia had been partially resolved, and the patient reported only mild pain at the surgery site with no other symptoms.

## Discussion

While several reports exist on mediastinal paragangliomas and the multifocal presentation of paragangliomas [12–19], only two other studies have documented multifocal presentations specifically associated with a mediastinal paraganglioma [20, 21]. The patient described in this study presented with multiple head and neck paragangliomas associated with a paraganglioma in the aortopulmonary prevascular space, a rare and unique manifestation of these tumors. This case provides valuable insights into the diverse clinical presentations of paragangliomas.

Various case reports have been reported multifocal parangangliomas affecting different regions of the body. Weinrach et al. reported a 58-year-old woman who was presented with two gangliocytic paragangliomas in the superior mediastinum and mid-distal esophagus [20]. In a case series study, Álvarez-Morujo et al. reported 24 patients with multifocal paragangliomas, two of whom had mediastinal masses. One patient was a 63-year-old female with a family history of paraganglioma, accompanied by a carotid body tumor and a jugular tumor. The other was a 36-year-old male with a carotid body tumor and a vagal paraganglioma. Both patients were found to have mutations in the succinate dehydrogenase gene. Although the authors acknowledged that surgical resection is the only definitive treatment for head and neck paragangliomas, they did not provide any information on how the mediastinal masses were managed [21]. Combining the findings from these studies, it appears that multifocal paragangliomas involving the mediastinum are quite rare. However, in patients with mediastinal paragangliomas, the possibility of involvement in other organs should be considered, necessitating further work-up to identify paragangliomas in other regions. Furthermore, these patients have a higher likelihood of being associated with genetic syndromes.

In the current case, the patient’s dysphonia was likely caused by left recurrent laryngeal nerve palsy due to compression by the tumor [22]. Moreover, we diagnosed the tumor just two months after symptoms began, allowing for timely intervention before it could invade the surrounding tissues. However, this might not be the case for all patients. Although mediastinal paragangliomas are extremely rare, they have a high mortality rate due to their tendency for local invasion and their proximity to critical organs, such as the heart and great vessels [23]. Therefore, timely diagnosis and management of these tumors is crucial. However, most patients remain asymptomatic, with symptoms primarily arising from catecholamine secretion in functional tumors, leading to issues such as tachycardia. In contrast, non-functional tumors typically cause symptoms due to mass effects, including hoarseness, dysphagia, and chest pain [24]. A variety of imaging techniques have been employed for the diagnosis of paragangliomas, including ultrasound, CT scan, MRI, positron emission tomography (PET) scan, and CT angiography [17, 25–27]. However, these modalities may have limitations in distinguishing paragangliomas from other tumors, which is critical given the risk of misdiagnosing paragangliomas, particularly when they occur in unusual locations [8]. One advantage of CT angiography over other imaging modalities is its ability to reveal the characteristic features of paragangliomas, such as the lightbulb sign, which aids in distinguishing paragangliomas from other tumors [27].

Given that paragangliomas are relatively resistant to chemotherapy and radiotherapy, and considering that surgery is the treatment of choice for paragangliomas in the anterior and middle mediastinum, we proceeded with surgical resection of the tumor [28]. The procedure was successful, as our patient was discharged without any surgery-related complications, and his symptoms were completely resolved. We could follow the patient for three months, and there was no recurrence during that period. Recurrence is also not common among people with mediastinal paraganglioma. In a study on 51 patients with paraganglioma, no recurrence occurred among the patients [29]. In another study, recurrence occurred in two patients with complete resection of the mass [30]. Among patients with multifocal paraganglioma, recurrence was observed in those who underwent surgery or received radiotherapy [21]. Therefore, given the substantial risk of recurrence and the limited knowledge of the clinical course of multifocal paragangliomas, lifelong surveillance using biochemical tests and imaging modalities is recommended.

One limitation of this case report was the lack of genetics evaluation of this patient. Given the tendency of multifocal paragangliomas to be linked with familial genetic disorders [9, 10] and the fact that the patient’s cousin also had a history of a neck mass, it is likely that the patient had a genetic predisposition to paragangliomas. Therefore, this situation warranted genetic evaluation and counseling to better understand the factors predisposing the patient to multifocal paraganglioma [31]. However, despite the significance of such an assessment, it was not performed due to the lack of resources and infrastructure for genetic testing in our country. This highlights a limitation in the proper management of patients with multifocal paraganglioma, particularly in the context of developing countries.

## Conclusion

This case report presents a rare manifestation of paraganglioma, characterized by multiple head and neck paragangliomas alongside a mediastinal paraganglioma. It offers valuable insights into the diverse presentations of these tumors. The study emphasizes the importance of conducting a comprehensive evaluation for patients with paragangliomas in atypical locations and underscores the need to consider potential genetic syndromes in this patient population.

## Data Availability

No datasets were generated or analysed during the current study.
